# Original Communication

**Published:** 1841-04-17

**Authors:** John C. Mercer


### ORIGINAL COMMUNICATION.

*Williamsburg, (Va.,) Feb.* 7, 1841.

To the Editors of the Medical Examiner.

*Gentlemen,*—It is my wish to make known to you a case of tubercular disease, which had an anomalous termination, and one to which I can find no parallel in authors. Its peculiarity may entitle it to a notice in your periodical. Perhaps your own experience, or your research, may furnish instances of like character.

The subject of the disease under consideration had for some time before her death manifested many of those symptoms which characterize the existence of a tendency to tubercular affections. It was pregnancy, which prevented its development at an earlier period. No sooner had parturition taken place, than the destructive malady presented many of its formidable features. Hectic fever and its train came on, with, however, no tangible evidences of the presence of pulmonary tubercles. The respiratory sounds manifested no other alteration than that which naturally occurred from the laboured and hurried movements of the pulmonary apparatus. Percussion betokened little or no change in the air-vesicles. Nevertheless, the præcordial distress, difficulty of respiration, general anxiety, and jactitation, were of the most strongly marked character. No relief was experienced from any remedy whatever.

Whence the origin of all this disturbance? The cause was readily perceived, when the ear was applied to the region of the heart. The first sound of this organ was nearly destroyed, so that it was scarcely distinguishable from the second cardiac tone. Dissection revealed that here was the source of all the distressing symptoms above mentioned. The tricuspid valve was almost obliterated by the presence of tubercular matter, which had maturated and softened. The lungs contained a few tubercles in the first stage, and so scattered were they through the parenchyma of these organs, as to defy detection even by the most experienced auscultators. The peritoneum exhibited these bodies abundantly disseminated on its surface, and presented, more than any other part, evidences of that tubercular diathesis, which was so forcibly manifested during life. Death taking place before the disease had reached that period of its progress at which emaciation becomes one of the prominent symptoms, the body showed no marks of reduction, either externally or on dissection. It may, therefore, be noted as an instance of acute phthisis.

This case is very interesting in another point of view. It proves to us the presence of a peculiar morbid diathesis, designated as the tubercular, and capable of throwing the system into that state known as the hectic condition, even before any foreign bodies, as tubercles, exist in such a degree and such a stage, as to account for the peculiar disturbances suffered by the economy. It cannot be supposed that the destruction of one of the valves of the heart could have caused the characteristic symptoms of hectic, had this affection not been connected with the existence of tubercles, for the loss of a valve's functions would only be expressed by marks significant of the improper distribution of the blood.

The utility of auscultation was beautifully exemplified in this instance of pathology. As a science, even considered devoid of all practical importance, it taught the observer to form a correct diagnosis, and consequently enlightened him very much in his prognosis. But it was in this case likewise of practical moment, inasmuch as it pointed to the heart as the organ needing the assistance of remedial agents. If percussion and auscultation, together with an examination of the external chest, and a strict scrutiny of the movements of the thoracic viscera as manifested to the eye, were steadily pursued in every case where the diagnosis is difficult and doubtful, we should have fewer charges against medicine as a science, and much feebler opposition from empyricism.

I beg leave, gentlemen, to make two or three more remarks not altogether connected with the preceding observations. How much is it to be regretted that in the practice of our profession, plain, common sense, should not be more generally consulted than is now the custom! Disease is an unnatural condition of some organ or organs, and not an indefinable something, against which we must wage war with our drugs. When called to a case, let us sit down quietly by our patient, and endeavour first to ascertain *what organ* is affected,—and, secondly, *how* it is affected. This can only be done by appropriate and numerous questions; and he who makes up his prescription at the mere sight of his patient, as though divinely inspired, can deserve no title but that of empiric. Yet how frequently is it the case that the sick are scarcely interrogated; and if they are, it is not with that point and narrow investigation, which the health, and, as far as human agency is concerned, the existence, perhaps, of the confiding sufferer require. When this is done, when it is seen by the world that we do not strive to shroud our doings in a cloud of mystery, and move against disease with an array of dark incantations, then, and then only, can we hope to triumph over quackery, and free our profession from a load of obloquy, which, it is be feared, it now too well merits.

With great respect, your obedient servant,
JOHN C. MERCER.

This case is very uncommon, so far as the disease of the tricuspid valve is concerned. The tendency to tuberculous disease, and the simultaneous deposit of granulations in the serous membranes and the lungs, are well known facts. It is only to be regretted that the author does not describe more fully the nature of the lesions of the tricuspid valve, and the condition of the rest of the heart, for it is not proven that the lesion of the valve is the cause of the absence of the first sound of the heart. The fever was undoubtedly the tuberculous fever which generally accompanies the formation of scattered granulations; we do not, however, regard this fever as precisely identical with the true hectic fever which occurs in the later periods of phthisis. It is the usual fever of acute phthisis; the peculiarity of the case is the disease of the tricuspid valve.—EDS.

